# Editorial Comment to Validation of schedules for optimal PSA monitoring after radical prostatectomy

**DOI:** 10.1111/iju.15397

**Published:** 2024-01-16

**Authors:** Kazuhiro Matsumoto

**Affiliations:** ^1^ Department of Urology Keio University School of Medicine Shinjuku‐ku Tokyo Japan

First and foremost, we extend our gratitude to Dr. Blas and colleagues for their invaluable validation of both our original and modified Keio PSA (prostate‐specific antigen) follow‐up schedule models.^1^ Radical prostatectomy stands as the primary treatment for clinically localized prostate cancer, with recurrence most commonly diagnosed through the detection of asymptomatic elevation in serum PSA levels. Given that serum PSA is solely produced by prostatic epithelial cells, it typically falls below 0.1 ng/mL postradical prostatectomy.^2^ Consequently, the persistent elevation or subsequent reincrease in serum PSA levels after surgery inevitably signals either residual cancer or recurrence. Therefore, postradical prostatectomy follow‐up is refreshingly straightforward, requiring only serum PSA measurements, without the need for imaging studies such as CT, MRI, or scintigraphy. In other words, unlike other forms of cancer, prostate cancer's postoperative follow‐up can be safely overseen by primary care clinics other than urologists.

Nevertheless, the most significant challenge remains determining the optimal timing for PSA measurements. During the early biochemical recurrence stage (PSA > 0.2 ng/mL), tumor cells may be confined to the pelvis, potentially making them more responsive to localized salvage radiation therapy. Considering that large‐scale retrospective cohort studies support the use of salvage radiation therapy in the early stages (PSA < 0.5 ng/mL),^3^ the prompt detection of PSA elevation in postoperative follow‐up is paramount.

Dr. Blas et al. proposed the Kyushu model for PSA monitoring after radical prostatectomy,^4^ which may prevent the detection of biochemical recurrence when PSA exceeds 0.4 ng/mL, compared to the original and modified Keio models. Meanwhile, it necessitates more frequent PSA measurements, which could be a potential limitation. In reality, it is impossible to eliminate oversight risks entirely, underscoring the need to establish a threshold for an acceptable oversight rate. To create the most effective follow‐up schedule model, we must carefully consider factors such as oncological benefits, medical costs, and clinical feasibility.

Nevertheless, these simple PSA follow‐up schedule tables are expected to serve as valuable tools in seamlessly transitioning patients to local clinics after radical prostatectomy, thereby reducing the medical burden on medical centers.

## CONFLICT OF INTEREST STATEMENT

None declared.
